# Efficacy of indoxacarb applied to cats against the adult cat flea, Ctenocephalides felis, flea eggs and adult flea emergence

**DOI:** 10.1186/1756-3305-6-126

**Published:** 2013-05-03

**Authors:** Michael W Dryden, Patricia A Payne, Vicki Smith, Kathleen Heaney, Fangshi Sun

**Affiliations:** 1 Department of Diagnostic Medicine/Pathobiology, Kansas State University, Manhattan, KS, 66506, USA; 2 Merck Animal Health, 556 Morris Avenue, Summit, NJ, 07901, USA

**Keywords:** Flea, Ctenocephalides felis felis, Cat flea, Cats, Indoxacarb, Egg production, Adulticidal activity, Efficacy

## Abstract

**Background:**

A study was conducted to evaluate the effect of indoxacarb applied to cats on adult cat fleas, *Ctenocephalides felis*, flea egg production and adult flea emergence.

**Methods:**

Sixteen cats were selected for the study and allocated to two treatment groups. Eight cats were treated with a 19.5% w/v topical spot-on solution of indoxacarb on day 0 and eight cats served as untreated controls. Each cat was infested with 50 fleas on Days -2, 7, 14, 21, 28, 35 and 42. On Days 1, 2, and 3, and at 2 and 3 days after each post treatment reinfestation flea eggs were collected from the pan under each cat cage. Eggs were counted and viability assessed by evaluating adult flea emergence 28 days after egg collection. Three days after treatment or infestation, each cat was combed to remove and count live fleas.

**Results:**

Treatment with indoxacarb provided 100% efficacy following infestations on day -2, 7, 14, 21 and 28 and efficacy was 99.6% following infestations on days 35 and 42. Egg production from indoxacarb treated cats was reduced by 99.9% within 72 hours of treatment. For subsequent infestations no eggs were produced from treated cats from day 8 through day 30. Egg production was still reduced by ≥95.8% through day 45. Indoxacarb treatment also reduced adult flea emergence from eggs for 5 weeks after treatment. The combination of reduction in egg numbers and egg viability from indoxacarb treated cats reduced predicted flea emergence by 100% from days 2 – 31 and 99.9%, 100%, 96.4% and 99.0% on days 37, 38, 44 and 45, respectively.

**Conclusions:**

A topical spot-on formulation of indoxacarb provided ≥99.6% efficacy against flea infestations on cats for 6 weeks following a single treatment. Indoxacarb also eliminated or markedly reduced egg production for the entire evaluation period and reduced the viability of the few eggs that were produced from Day 1 through Day 38. Given indoxacarb’s effect on adult fleas, egg production and egg viability; this formulation can interrupt flea reproduction on treated cats for at least 6 weeks after treatment.

## Background

The cat flea, *Ctenocephalides felis*, is the most important ectoparasite of dogs and cats in most areas of the world [1]. Once cat fleas acquire a host, they mate and start laying eggs within 24 – 48 hours [2,3]. Fleas deposit their eggs into the haircoat of the dog or cat with the eggs dropping off into the premises where within a few weeks they develop into adult fleas [3]. Since female cat fleas can produce 40 – 50 eggs/day it does not take long for massive numbers of flea eggs and ultimately emerging fleas to accumulate within the premises [3].

Currently, most flea control is based on residual topical spot-on treatments or oral medications [4-6]. The primary focus of these topical and systemic flea control efforts is to force fleas into “extinction” in a localized environment (home or yard) by preventing reproduction [6,7]. Eradication of an existing flea infestation is accomplished either by killing newly acquired fleas with a residual adulticide before they can initiate reproduction and/or affecting the viability of the eggs they produce through the use of insect growth regulators or other ovicidal compounds [4]. In order for a topical or systemic insecticide to prevent egg production, the residual activity must be sufficient to kill or render moribund newly acquired fleas within 24 hours. A number of in-home and multicentric studies on three continents have demonstrated that this approach to flea control can be successful [6,8-13].

Indoxacarb is a novel oxadiazine insecticide that controls a broad spectrum of insects and was originally developed for use on a variety of plants including vegetables, fruit trees, cotton and row crops [14]. Indoxacarb is a pro-insecticide that is metabolized within the insect to a more active form: an N-decarbomethoxylated metabolite of indoxacarb. This metabolic conversion of indoxacarb, known as bioactivation, is attributed to actions of esterase and amidase enzymes within the insect [15,16]. The active metabolite of indoxacarb exerts its effect by blocking the voltage-gated sodium ion channels in insects and is at least forty times more potent than parent indoxacarb in its ability to block sodium channel ion current [15-17]. Mammals exhibit minimal bioactivation of indoxacarb and sodium channel binding studies in rats show the active indoxacarb metabolite has much weaker potency against mammalian sodium channels than insect channels [16,18].

This study was undertaken to evaluate the efficacy of a recently developed spot-on formulation of indoxacarb applied to cats against adult fleas, flea egg production and development of fleas from eggs to adults.

## Methods

### Animals and housing

Nineteen (10m:9f; > 6mo; 2.7 to 4.2 kg), purpose bred domestic shorthair cats were used in this study. Cats used in this investigation had no exposure to ectoparasiticides prior to treatment and were in good health throughout the study. Cats were housed individually in standard stainless cages steel with solid sides and back, with a steel barred door. Cats were physically separated by the solid stainless steel cage walls. Cats were fed a commercial dry cat-food ration. Water was available ad libitum. Physical examinations were conducted by a licensed veterinarian prior to the initial flea infestation and cats were determined to be in good health and free of any preexisiting dermal lesions. All animal care procedures conformed to guidelines established by the Institutional Animal Care and Use Committee at Kansas State University (Approval No. 3124).

### Animal selection and randomization

On Day -8, all cats were infested with 50 adult cat fleas, *C. felis*, (KS1 strain) 1 to 5 days post emergence. On Day -5, flea comb counts were performed to evaluate the susceptibility of each cat to maintain experimental infestations and for random allocation of the cats to the treatment groups. Cats were combed with a fine-toothed flea comb having 12 to 13 teeth/cm. Flea removal was achieved by combing each cat thoroughly for 10 minutes. If five or more fleas were recovered during this period, the cat was combed for an additional 5 minutes. If any fleas were recovered during the second combing period, the cats were combed for an additional 5 minutes. Personnel conducting flea combing and flea counts were blinded to treatment groups. The 16 cats (7m:9f) with the highest pre-treatment flea counts were used for the study. Within gender, cats were ranked from highest to lowest flea count, with the female cat with lowest flea number placed in male gender grouping, and blocked in two groups containing 8 cats each.

### Treatments

Cats (4m:4f) in treatment group 1 were treated topically with indoxacarb (2 – 4.0 kg were treated with 0.51 mL and cats > 4.1 kg were treated with 1.03 mL indoxacarb, 19.5% w/v; ACTIVYL® Merck Animal Health) applied using the commercial product pipette or a syringe. Treatment was administered by parting the hair with the tip of the pipette or syringe at the base of the skull of the cat and the entire contents were applied directly to the skin. Cats (3m:5f) in treatment group 2 served as untreated controls. Cats were observed following treatment for any adverse events associated the treatments.

### Efficacy evaluations

On Days -2, 7, 14, 21, 28, 35, and 42, each cat was infested with approximately 50 unfed cat fleas, *C. felis* (KS1 strain). On Days 1, 2, and 3, and at 2 and 3 days after each post treatment reinfestation flea eggs were collected from the pan under each cat cage. Prior to the egg collections, cats were massaged/brushed vigorously by hand for ~20 seconds in their cages to dislodge any flea eggs from the cat’s hair coat, allowing the eggs to fall into the drop pan below the cage. Eggs collected were counted by placing the eggs in a glass Petri dish and counted under a dissecting microscope. At approximately 72 hours after treatment or infestation, each cat was combed to remove and count live fleas. Viability of eggs was determined by placing up to 100 eggs into glass Petri dishes containing flea growth media and holding in a rearing chamber (I-30B, Percival Manufacturing Co., Boone, IA) at 27-28°C, 70-80% relative humidity, 24 hours dark. If less than 100 eggs were collected, the entire sample was placed into the Petri dish containing flea growth media and similarly processed. At 10 – 12 days after egg collection, pupae (and any larvae that had not completed cocoon formation) were sifted from the media and placed into plastic vials with lids. Adult emergence was determined by counting adult fleas at 28 days after egg collection. Personnel conducting comb counts, egg collections and viability assessments were blinded to treatment group allocation.

### Data analysis

Data at each time point were analyzed separately. The adult flea and egg count data were transformed prior to analysis using the Y = log_e_(x + 1) transformation. The log transformed data were analyzed by a mixed linear model including treatment as the fixed effect; and block as the random effect. Least squares means were used for treatment comparisons and were back transformed to obtain the estimates of geometric mean flea and egg counts. Percentages of the emerging adult flea count were analyzed by a mixed linear model including treatment as the fixed effect; and block as the random effect. A two-tailed test was used for the comparison between groups. Statistical significance was declared when p < 0.05. The primary software was SAS version 9.3.

Percent control of adult fleas was calculated using geometric means with Abbott’s formula:

$$\mathrm{Efficacy}\left(\%\right)=100\times \left(\mathrm{MC-MT}\right)/\mathrm{MC}$$

Where:

- MC is the geometric mean number of total adult live fleas on untreated cats

- MT is the geometric mean number of total adult live fleas on treated cats.

The percent control of egg production and egg viability were also calculated using Abbott’s formula by adjusting the mean number of eggs produced and mean percent of emergent fleas from treated cats with the mean number of eggs produced and mean percent of emergent fleas produced by fleas on untreated control cats.

## Results

All cats included in the study demonstrated adequate pretreatment flea retention with day -5 flea counts ranging from 16 – 38 fleas/cat. Nontreated cats also maintained adequate flea infestations throughout the study with geometric mean flea counts ranging from 19.3 to 28.0 (Table 1).

**Table 1:** Geometric mean adult flea counts and percent efficacy relative to non-treated controls for cats treated with a 19.5% w/v indoxacarb topical spot-on

**Study day**	**Mean # of Fleas**^**1,2**^	
	**Non-treated controls**^**3**^	**Indoxacarb**^**3**^
3	27.2 ^a^	0.0 ^b^ (100.0)^4^
10	19.3 ^a^	0.0 ^b^ (100.0)
17	25.6 ^a^	0.0 ^b^ (100.0)
24	28.0 ^a^	0.0 ^b^ (100.0)
31	24.7 ^a^	0.0 ^b^ (100.0)
38	25.7 ^a^	0.1 ^b^ (99.6)
45	20.8 ^a^	0.1 ^b^ (99.6)

^1^ Each cat was infested with 50 adult *Ctenocephalides felis* from the KS1 strain on days -2, 7, 14, 21, 28, 35 & 42.

^2^Geometric mean # of fleas recovered from cats per treatment group.

^3^Each of 8 cats in the control group received no treatment. Each of 8 cats in the 19.5% w/v indoxacarb group were administered the topical spot-on according to label directions on Day 0.

^4^% efficacy = ((geometric mean live adult flea count control-geometric mean live adult flea count treatment)/geometric mean live adult flea count control) × 100.

^a, b^ geometric means across rows with unlike letter superscripts are significantly different (P < 0.001).

Cats in the indoxacarb treatment group were administered an average of 33.7 mg/kg (27.3 - 47.8 mg/kg) indoxacarb. This single dose of indoxacarb produced highly significant reductions in geometric mean flea counts on treated cats throughout the entire 45 days of the study (P < 0.001, Table 1). Treatment with indoxacarb provided 100% efficacy following infestations on day -2, 7, 14, 21 and 28 and 99.6% reduction in geometric mean flea counts following infestations on days 35 and 42 (Table 1).

Geometric mean flea egg counts for the indoxacarb-treated cats were also significantly lower than those for nontreated controls at all post-treatment evaluations (P < 0.001, Table 2). Egg production from indoxacarb treated cats was reduced by 66.2% within 24 hours after treatment and 99.9% within 72 hours of treatment. For subsequent infestations egg production was non-existent from treated cats out to day 30 and 99.9% on day 31 relative to nontreated cats (Table 2). Egg production was still markedly reduced by 95.8% and 98.7% on days 44 and 45, respectively. The geometric mean number of eggs produced from fleas on control cats from day 9 through day 45 post-treatment ranged from 229.7 - 1,086.1. Whereas, the geometric mean number of eggs from indoxacarb treated cats was highest on day 44 at 9.7 eggs/cat (Table 2).

**Table 2:** Geometric mean flea egg counts and percent efficacy relative to non-treated controls for cats treated with a 19.5% w/v indoxacarb topical spot-on

	**Non-treated controls**^**1,2**^	**Indoxacarb**^**1,2**^	
**Day of egg collection**	**Mean # of eggs**^**3**^	**Mean # of eggs**	**Efficacy**^**4**^
1	654.2 ^a^	220.9 ^a^	66.2
2	627.8 ^a^	7.2 ^b^	98.9
3	589.8 ^a^	0.6 ^b^	99.9
9	650.7 ^a^	0.0 ^b^	100.0
10	957.6 ^a^	0.0 ^b^	100.0
16	459.1 ^a^	0.0 ^b^	100.0
17	686.4 ^a^	0.0 ^b^	100.0
23	739.8 ^a^	0.0 ^b^	100.0
24	1086.1 ^a^	0.0 ^b^	100.0
30	566.5 ^a^	0.0 ^b^	100.0
31	732.1 ^a^	0.1 ^b^	99.9
37	491.2 ^a^	0.7 ^b^	99.9
38	577.9 ^a^	0.1 ^b^	99.9
44	229.7 ^a^	9.7 ^b^	95.8
45	508.4 ^a^	6.8 ^b^	98.7

^1^Each of 8 cats in the control group received no treatment. Each of 8 cats in the 19.5% w/v indoxacarb group were administered the topical spot-on according to label directions on Day 0.

^2^ Each cat was infested with 50 adult *Ctenocephalides felis* from the KS1 strain on days -2, 7, 14, 21, 28, 35 & 42.

^3^Geometric mean # of eggs recovered from cats per treatment group.

^4^% efficacy = ((geometric mean # eggs control-geometric mean # eggs treatment)/geometric mean # eggs control) × 100.

^a,b^ Geometric means across rows with unlike letter superscripts are significantly different (P < 0.001).

Based on percent adult emergence, indoxacarb treatment also had an apparent lethal effect on the viability of flea eggs collected from treated cats on days 1–3 and on days 31 and 38 (P < 0.001, Table 3). Since no eggs were collected from indoxacarb treated cats from days 9 – 30 no percent adult flea emergence analysis could be conducted. A direct effect on egg viability based on percent adult emergence was not observed on post-treatment days 44 and 45. However when egg viability is considered along with the marked reduction in egg numbers, indoxacarb did demonstrate a marked effect upon the reproductive success of the fleas even at post-treatment day 45 (Table 3). The combination of the geometric mean number of eggs produced and the percent adult emergence from those eggs results in the predicted adult emergence or reproductive success. The predicted adult emergence from eggs produced by fleas on the untreated control cats ranged from 177.5 – 908.2 emerging fleas/cat. Whereas the reduction in predicted flea emergence from indoxacarb treated cats was 100% from days 2 – 31 and was still 96.8% and 99.0% on days 44 and 45, respectively (Table 3).

**Table 3:** Geometric mean percent adult emergence, predicted adult emergence and percent efficacy relative to non-treated controls for predicted adult flea emergence from eggs collected from cats treated with a 19.5% w/v indoxacarb topical spot-on

	**Non-treated controls**^**1,2**^		**Indoxacarb**^**1,2**^		
**Study day**^**3**^	**% Adult emergence**^**4**^	**Predicted emergence**^**5**^	**% Adult emergence**^**4**^	**Predicted emergence**^**5**^	**Efficacy**^**6**^
1	50.1^a^	327.9	4.6 ^b^	10.2	96.9
2	46.1^a^	289.6	0.0^b^	0.0	100.0
3	67.0^a^	395.2	0.0^b^	0.0	100.0
9	77.1	501.8	NE	0.0	100.0
10	79.8	763.7	NE	0.0	100.0
16	75.3	345.5	NE	0.0	100.0
17	76.4	524.2	NE	0.0	100.0
23	84.3	623.2	NE	0.0	100.0
24	83.6	908.2	NE	0.0	100.0
30	77.0	436.2	NE	0.0	100.0
31	85.2^a^	604.0	0.0^b^	0.0	100.0
37	75.4^a^	370.2	20.0^b^	0.1	99.9
38	63.7^a^	388.6	0.0^b^	0.0	100.0
44	77.3^a^	177.5	66.1^a^	6.4	96.4
45	82.3^a^	418.1	62.5^a^	4.3	99.0

^1^ Each of 8 cats in the control group received no treatment. Each of 8 cats in the 19.5% w/v indoxacarb group were administered the topical spot-on according to label directions on Day 0.

^2^ Each cat was infested with 50 adult *Ctenocephalides felis* from the KS1 strain on days -2, 7, 14, 21, 28, 35 & 42.

^3^ Days post-treatment that eggs were collected.

^4^ % Adult emergence = (# of adults emerging/# of eggs collected) × 100.

^5^ Predicted emergence = (% emergence × geometric mean # eggs collected from Table 2).

^6^ % Efficacy = ((predicted adult emergence control-predicted adult emergence treatment)/geometric mean % adult emergence control) × 100.

NE = no flea eggs collected.

^a,.b^ Values across rows with unlike superscripts are significantly different (P < 0.05).

There were no adverse events associated with indoxacarb treatments in this study.

## Discussion

Veterinarians and pet owners should expect that when correctly applied indoxacarb spot-on will control the existing flea burden on a cat and that there should be a rapid reduction in egg production from the existing flea infestation. Within 48 hours of application egg production was almost completely halted (98.9% reduction). Since none of those eggs produced adult fleas no dissemination of viable eggs occurred from treated cats within 2 days of treatment.

Most currently available residual topical spot-on and systemic insecticide formulations are marketed to provide at least 30 days of effective flea control. Indoxacarb spot-on certainly provided and exceeded that level of control. A single application of indoxacarb provided ≥99.6% control of adult cat fleas on cats for at least 45 days after treatment.

Following treatment, egg production from weekly flea reinfestations was reduced by ≥99.9% at least 5 weeks. Consumption of blood is necessary before cat fleas can initiate reproduction and egg production does not begin until 24–48 hours after females take their first blood meal [1,2]. Therefore, if a residual insecticide can kill or produce toxicity in newly acquired fleas within 24 hours, egg production should be markedly reduced or halted. While this study did not evaluate the residual speed of kill of indoxacarb or reduction of blood consumption by adult fleas, it did demonstrate that indoxacarb had a profound effect upon egg production. This indirectly indicates that indoxacarb is rapidly toxic to fleas and thus markedly reduces blood feeding by fleas.

Indoxacarb also had an effect upon adult flea emergence from eggs. While this study did not evaluate egg hatch directly it is anticipated that the effect of indoxacarb was on egg viability. Eggs collected from trays under treated cats were sorted and removed from all debris from those cats. The eggs were subsequently placed into standard rearing media. Following this procedure any larvae hatching from those eggs would have had no further contact with indoxacarb. Similar marked lethal effect on egg viability by a flea adulticide has been previously observed with selamectin [19].

Effective residual flea adulticides should have sufficient residual activity to provide for continuing kill of most if not all the newly emerging fleas that jump onto the treated pet between applications. If a flea product also has an effect upon the viability of the eggs produced by any female flea surviving the adulticidal effect, that product would markedly suppress the reproductive success of a flea population [6,7]. In this study indoxacarb demonstrated marked flea kill, profound reduction in egg production and apparent ovicidal activity, as has been observed in other insect species [15]. This combination of attributes provided ≥99.9% reduction in reproductive success (predicted emergence) for 5 weeks following a single application of indoxacarb. Proper application of indoxacarb to cats in a flea infested home should effectively drive the flea population to extinction.

This study was conducted using the KS1 flea strain. Several previous studies have demonstrated that this strain has reduced susceptibility or outright resistance to carbaryl, chlorpyriphos, fenthion, fipronil, imidacloprid, permethrin, pyrethrins, and spinosad [3,19-24]. This study showed that indoxacarb performed well against this KS-1 flea strain.

## Conclusions

A topical spot-on formulation of indoxacarb provided 100% efficacy against flea infestations on cats for 4 weeks following a single treatment and was still providing 99.6% reduction in flea counts through week 6. Indoxacarb eliminated or markedly reduced egg production for the entire evaluation period. The formulation also reduced the viability of the few eggs that were produced through Day 38. Given indoxacarb’s effect on adult fleas, egg production and egg viability; it appears this formulation can interrupt flea reproduction for at least 6 weeks after treatment.

## Competing interests

MWD has had research funded and has been sponsored to lecture by numerous pharmaceutical companies, including Merck Animal Health. Kathleen Heaney and Fangshi Sun are employees of Merck Animal Health.

## Authors’ contributions

MWD assisted in the design of the study, served as study investigator and drafted the manuscript. VS & PAP coordinated and supervised data collection and entry and revision of manuscript; KH assisted in design of study, monitoring of study and manuscript revision. FS conducted the statistical analysis of the data. All authors reviewed and approved the final manuscript.
